# Concurrent alteration in inflammatory biomarker gene expression and oxidative stress: how aerobic training and vitamin D improve T2DM

**DOI:** 10.1186/s12906-022-03645-7

**Published:** 2022-06-22

**Authors:** Rastegar Hoseini, Hiwa Ahmed Rahim, Jalal Khdhr Ahmed

**Affiliations:** 1 Department of Exercise Physiology, Faculty of Sport Sciences, Razi University, Kermanshah, Iran; 2 Physical Education and Sport Sciences Department, University of Halabja, Halabja, 46018 Kurdistan Region Iraq

**Keywords:** Aerobic training, Vitamin D supplementation, Signaling pathway, Inflammation, Oxidative stress

## Abstract

**Background:**

Vitamin D (Vit D) supplementation and Aerobic Training (AT) exert several beneficial effects such as antioxidant and anti-inflammatory actions. The literature on the effects of AT and Vit D supplementation on the oxidative stress biomarkers and gene expression of inflammatory cytokines in patients with Type 2 Diabetes Mellitus (T2DM) is limited. The present study aimed to examine the effects of AT and Vit D supplementation on inflammation and oxidative stress signaling pathways in T2DM patients.

**Materials and methods:**

In this single-blinded, randomized, placebo-controlled trial, 48 men with T2DM (aged 35–50 years with Body Mass Index (BMI) of 25–30 kg/m2) were randomly allocated into four groups: AT+Vit D (*n* = 10); AT + placebo (AT; *n* = 10); Vit D (*n* = 10), and Control + placebo (C; *n* = 10). The eight-week AT program was executed for 20–40 min/day, at 60–75% of heart rate maximum (HRmax), for 3 days/wks. The Vit D group received 50,000 IU of Vit D supplement capsules per week for 8 weeks. The serum levels of oxidative stress biomarkers and gene expression of inflammatory cytokines in the Peripheral Blood Mononuclear Cells (PBMCs) were evaluated using the RT-PCR method. To analyze the data, paired t-tests and one-way analysis of variance and Tukey’s post hoc test were used at the significance level of *P* < 0.05.

**Results:**

The result shows that serum 25-OH-Vit D, total nitrite, Total Glutathione (GSH), Total Antioxidant Capacity (TAC), Superoxide Dismutase (SOD), Catalase (CAT), and Glutathione Peroxidase (GPX) increased; and insulin, Fasting Blood Glucose (FBG), Homeostasis Model Assessment of Insulin Resistance (HOMA-IR), High Sensitivity C-Reactive Protein (hs-CRP), Malondialdehyde (MDA), glycated albumin, and Urinary 8-hydroxydeoxyguanine (8-OHdG) decreased significantly in all groups after 8 weeks, except for C. In addition, results of RT-PCR showed that AT+Vit D, Vit D, and AT significantly downregulated the gene expression of Tumor Necrosis Factor-Alpha (TNF-α), Interleukin-1 Beta (IL-1β), Mitogen-Activated Protein Kinases 1 (MAPK1), Nuclear Factor Kappa B (NF-κB) 1 (p50). It also upregulated Interleukin-4 (IL-4) gene expression, Peroxisome Proliferator-Activated Receptor Gamma (PPAR-γ) in T2DM patients compared to the C.

**Conclusion:**

Additionally, the AT+Vit D group showed significantly lower insulin, FBG, HOMA-IR, hs-CRP, MDA, glycated albumin, urinary 8-OHdG, IL-1β, TNF-α, MAPK1, and NF-κB1 (p50) levels and significantly higher serum 25-OH-Vit D, total nitrite, GSH, TAC, CAT, SOD, GPX, IL-4, and PPAR-γ levels compared to the AT and Vit D groups. In T2DM patients, 8 weeks of AT+Vit D had a more significant impact on certain gene expressions related to inflammation and oxidative stress than Vit D or AT alone.

## Introduction

Type 2 Diabetes Mellitus (T2DM) is a chronic disorder leading to hyperglycemia resulting from abnormal insulin function and secretion. According to the World Health Organization (WHO), the number of T2DM patients is expected to reach 642 million by 2040 [1]. Increased inflammatory cytokine is an inseparable part of T2DM and is associated with protein-energy malnutrition, cardiovascular disease (CVD), and all-cause mortality [2]. Increased inflammatory biomarkers, such as Tumor Necrosis Factor-Alpha (TNF-α), Interferon Gamma (IFN-g), and IL-1 Beta (IL-1β), Mitogen-Activated Protein Kinases 1 (MAPK1), were associated with T2DM [3, 4]. Furthermore, various factors, including lack of antioxidant vitamins and microelements, increased oxygen metabolism, inflammatory factors, and uremic toxicity, would result in oxidative stress in patients with T2DM [5]. Lifestyle alterations, i.e., exercise and supplementation, are complementary treatment approaches in T2DM patients [6]. Evidence has indicated that Aerobic Training (AT) exercise intervention is strongly recommended for T2DM patients to boost weight loss, improve lipid profile, facilitate skeletal muscle glucose uptake, and increase the abundance of Glucose Transporter Type 4 (GLUT4) [7, 8]. According to Teixeira de Lemos’s review, regular moderate-intensity exercise, as a natural antioxidant and anti-inflammatory strategy, helped control oxidative stress and inflammation in diabetic Zucker Diabetic Fatty (ZDF) rats [9]. While acute or exhaustive exercise can be damaging, several studies showed positive alteration of the oxidative homeostasis of cells and tissues following regular exercise training, possibly via decreasing the basal levels of oxidative damage and increasing resistance to oxidative stress [10–14]. Nishida et al. demonstrated upregulation of antioxidant defenses in animal models of T2DM, increased Cu/Zn- Superoxide Dismutase (SOD) protein production after low-intensity exercise, and increased Mn-SOD as a result of moderate-intensity exercise [15]. Besides, other clinical and experimental studies also associated the exercise-induced reduction in oxidative stress with the increased nitric oxide bioavailability, endothelial nitric oxide synthase (eNOS) expression, and/or eNOS Ser phosphorylation consequent lower reactive oxygen species generation [14, 16, 17]. A single bout of exercise induces a transient increase in high sensitivity C-reactive protein (hs-CRP). However, regular moderate-intensity exercise increases anti-inflammatory biomarkers (e.g., Interleukin (IL); IL-4 and IL-10) [18], and decreases of hs-CRP, IL-6, and TNF-α protein content [19] that reinforces the anti-inflammatory characteristics of long-term exercise [20].

Furthermore, literature also showed the significant prevalence of hypovitaminosis D in T2DM patients, and both conditions are on the rise globally [21, 22]. Vit D has been linked to the development of T2DM, presumably by altering insulin secretion and, as a result, hyperglycemia [22, 23]. Hypovitaminosis D elevates the intracellular calcium levels by increasing the secretion of parathyroid hormone, which in turn impairs GLUT4 and inhibits the calcium-related insulin secretion and action [24]. There is limited literature on how Vitamin D (Vit D) affects gene expression related to oxidative stress biomarkers and inflammatory cytokines. Choi et al. (2013) reported reduced TNF-α and IL-4 gene expressions after treating rats with Vit D [25]. Additionally, calcitriol is known to inhibit TNF-α releasement in a dose-dependent manner [26]. In vitro animal studies have demonstrated that Vit D suppresses the activation of TNF-α-induced nuclear factor kappa B (NF-κB) and upregulates IL-4 through Vit D receptors (VDR) [27].

However, Carrillo et al. (2012) showed no significant alteration in TNF-α and IL-4 following a 12-week resistance training and high-dose Vit D in healthy overweight individuals [28]. The molecular mechanisms of AT and Vit D modulating inflammatory cytokines and oxidative stress are unknown. Peroxisome Proliferator-Activated Receptor Gamma (PPARγ) and Mitogen-Activated Protein Kinase 1 (MAPK1) participate in signaling cascades induced by pro-inflammatory factors. In addition, NF-κB is hypothesized to control pro-inflammatory cytokines in cellular responses [29].

To our knowledge, controversial and limited data exist on the effects of AT and Vit D supplementation on the oxidative stress biomarkers and inflammation gene expression in T2DM. This study evaluated the effects of Vit D and AT on the oxidative stress biomarkers and gene expression of inflammatory cytokines in T2DM patients.

## Methods

### Experimental approach

This study was a single-blinded, placebo-controlled, randomized clinical trial among 40 T2DM patients aged 35–50 years, registered on the Iranian clinical trial website at http://www.irct.ir:IRCT20210811052151N1 on 01/09/2021. This research was conducted according to the Declaration of Helsinki. The Research Ethics Committees approved the study protocol of Kermanshah Razi University (IR.RAZI.REC.1400.044); informed consent was taken from all patients.

### Subjects

**Fig. 1:**
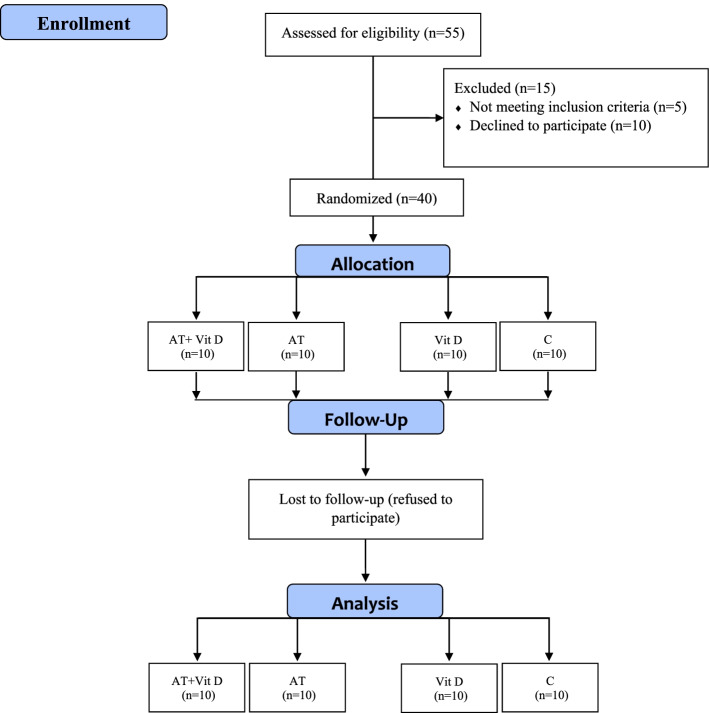
Flow chart of the study population

The exclusion criteria were as follows: having a regular exercise program that might confound the results of the AT program; history of heart disease, hypertension, orthopedic disorders, and smoking; COVID-19 infection and muscle injuries during the study that enable subjects to perform the exercise training program; taking Vit D, antioxidant and anti-inflammatory supplements (e.g., omega-3 fatty acids, vitamins E and C) and immunosuppressive medications in the past 3 months before participating in the study that might affect the variables of the present study or confound the results. The 36-subject sample size was estimated for this study using G. POWER 3.1 software (power of 0.99, alpha error of 0.05, and effect size of 0.85). Given the probability of participants’ refusal, 40 middle-aged (35–50 years) males with T2DM were selected randomly from the Baxshin Medical & Health Center, Sulaymaniyah, Iraq. Then, using the Random Number Generator approach, they were randomly allocated to four groups: AT+Vit D (*n* = 10), AT+placebo (AT; *n* = 10), Vit D (*n* = 10), and Control+placebo (C; *n* = 10), all of which had the same chance of being chosen (Fig. 1).

### Intervention

#### Aerobic training

The participants in the AT group were required to exercise at home three times a week for 8 weeks. All of the training protocols were supervised by the exercise physiologists.

According to the American Diabetes Association’s recommendation, the subjects were instructed to do AT for 20 minutes at 60% heart rate maximum (HRmax) per session, progressing to 40 minutes at 75% HRmax per session (ADA) [30] (Table 1). Also, 10 minutes of warming up and 10 minutes of cooling down were performed every session., The HRmax formula was used [HRmax = 220 − age] to determine the target heart rate [31]. Before the first training session, the participants learned the pulse palpation method to count their pulse and heart rates. Furthermore, the 6–20 Rating of Perceived Exertion (RPE) scale was utilized to ensure that the appropriate heart rate (exercise intensity) was obtained and sustained [32] (Table 1).

**Table 1:** The exercise program

Variables	Weeks							
	1st	2nd	3rd	4th	5th	6th	7th	8th
**Duration (min)**	20	25	30	30	35	35	40	40
**Intensity (HR**_**Max**_**)**	60–65%	60–65%	60–65%	65–70%	65–70%	65–70%	70–75%	70–75%
**RPE**	10	10	11	11	12	12	13	13

#### Supplementation

In the present study, Vit D and AT+Vit D received 50,000 IU/week of Vit D supplements (Zahravi Pharmaceutical Company, Tabriz, Iran), and AT and C received a placebo with the same color, taste, and shape (Barij Essence Pharmaceutical Company, Kashan, Iran). Additionally, 3-day food records (nutritionist IV; San Bruno, First Databank, CA) and physical activity levels were measured before the intervention in weeks 2, 4, 6, and 8.

### Measurements

#### Anthropometric and body composition

The participants were familiarized with the study method 3 days before the beginning and end of the intervention, and anthropometric variables and body composition were measured. Height was measured using a stadiometer (DETECTO, USA; Model 3PHTROD-WM) and waist circumference with a non-elastic tape measure to the nearest 0.5 cm. The bioelectric impedance analysis (Zeus 9.9 PLUS; Jawon Medical Co., Ltd., Kungsang Bukdo, South Korea) was used to assess Body Mass Index (BMI), Waist–hip ratio (WHR), Body Fat Percentage (BFP), and Bodyweight (BW) at 8–9 A.M. after at least 12-hour fasting. The participants were requested to refrain from taking diuretics and participating in intensive exercise activities 48 hours before the tests.

#### Outcomes

The primary outcome included the difference in the mean of inflammatory markers, including TNF-α, IL-1β, MAPK1, NF-κB1 (p50), IL-4, and PPAR-γ. The secondary outcome included insulin, Fasting Blood Glucose (FBG), Homeostasis Model Assessment of Insulin Resistance (HOMA-IR), serum 25-OH-Vit D, and oxidative stress biomarkers, such as Total Glutathione (GSH), Total Antioxidant Capacity (TAC), SOD, Catalase (CAT), and Glutathione Peroxidase (GPX), hs-CRP, Malondialdehyde (MDA), glycated albumin, and Urinary 8-hydroxydeoxyguanine (8-OHdG).

#### Biochemical assessment

Fasting insulin and glucose levels were evaluated by Enzyme-Linked Immunosorbent Assay (ELISA) (Sweden, Mercodia kits) and the enzymatic method (Iran, Pars Azmun Kit), respectively. The insulin resistance index was calculated using the HOMA-IR equation as follows: resistance (HOMA) = [glucose (mg/dL) × insulin (μU/mL)]/405 [33]. Moreover, the ELISA kits were used to determine serum 25-hydroxyvitamin D concentrations (IDS, Boldon, UK), serum hs-CRP concentration (LDN, Nordhorn, Germany) and plasma 8-OHdG (StressXpress ELA Kit, StressMarq Biosciences, Canada). An albumin assay reagent (Lucica GA-L kit, Asahi Kasei Pharma Corporation, Japan) determined serum albumin levels enzymatically. Also, the spectrophotometer was used to assess the SOD (Assay Kit, ZellBio GmbH, Ulm, Germany), CAT (Sigma-Aldrich kit, USA), MDA (Assay Kit, ZellBio GmbH, Ulm, Germany), GPX (Assay Kit, ZellBi, Germany), total nitrite (ZellBio assay kit, Germany), GSH (ZellBio assay kit, Germany), and TAC (Assay Kit, ZellBio, Germany).

#### Isolation of PBMCs

We examined the gene expressions associated with inflammation in Peripheral Blood Mononuclear Cells (PBMCs) from blood samples, which are the most available tissue for gene expression research, providing more information than plasma concentrations [34]. Before and after the study, 15 ml of fasting blood were drawn. For PBMCs isolation, 4 ml of blood was mixed through 3-part diluted blood to 2-part Ficoll-Hypaque and centrifuged for 30 min at 500×g, 30°. Then, 10 mL of phosphate-buffered saline (PBS) was added and centrifuged for 10 minutes at 400 g, 4C, followed by Hanks balanced salt solution and Percoll. After centrifuging for 25 minutes at 370 g, 25 °C, the cloudy layer in the top 5 mm was transferred into a separate tube using a sterile Pasteur pipet for further analysis.

#### RNA extraction and real-time PCR

Total RNAs were extracted from 5 mL peripheral blood with the trizol reagent (Invitrogen, USA) using an RNX-plus kit, Cinnacolon, Tehran, Iran. The RNA was quantified using a UV spectrophotometer, which revealed no contamination with protein or DNA (OD 260/280 ratio between 1.7 and 2.1), followed by reverse transcription to the cDNA library (via Moloney murine leukemia virus reverse transcriptase). Then, by using glyceraldehyde-3-phosphate dehydrogenase primers as housekeeping gene and quantitative RT-PCR method (LightCycler technology, Roche Diagnostics, Rotkreuz, Switzerland; SYBR green detection and Amplicon Kit), the gene expression of IL-1β, MAPK1, IL-4, PPAR-γ, NF-κB1 (p50), and TNF-α were evaluated. Additionally, the Primer Express (Applied Biosystems, Foster City) and Beacon designer (Takaposizt, Tehran, Iran) software were used to design the primers and Pffafi or 2 − 11CT method to calculate relative transcription levels (Table 2).

**Table 2:** Primers for Real-Time Quantitative PCR

Genes	Primers	Size (bp)	Annealing temperature (C)
IL-4	F: CTCACAGAGCAGAAGAACAC R: TGGTTGGCTTCCTTCACAG	223	60
MAPK1	F: GGAACAGCACCTCCACTATTT R: GCCACAATGTCTGCGTATCT	226	60
NF-kβ	F: GCTGAGTCCTGCTCCTTC R: GTCTATTTGCTGCCTTGTGG	208	59
TNF-α	F: GAGCCAGCTCCCTCTATTTATG R: CTACATGGGAACAGCCTATTGT	189	60
PPAR-γ	F: ATGACAGACCTCAGACAGATTG R: AATGTTGGCAGTGGCTCAG	210	55
IL1-β	F: GATGGCTTATTACAGTGGCAATG R: AGTGGTGGTCGGAGATTCG	137	56

### Statistical analysis

The data were analyzed by version 26.0 of SPSS software. The Shapiro–Wilk test was used to check the normality of the distribution in the continuous variables. The paired sample t-test was used for Within-group comparisons. Also, one-way ANOVA and Tukey’s posthoc were used for between-groups comparison at *P* < 0.05.

## Results

Table 3 shows the results of the mean ± SD of the anthropometric variables. After 8 weeks, the t-test results indicate significant differences in the WHR, BMI, BW, and BFP. The variables mentioned above decreased significantly in all intervention groups but increased significantly in the C. Accordingly, a significant difference was found between the AT+Vit D, AT, and Vit D groups and the C group in terms of BW, BMI, BFP, and WHR. A significant difference was also observed in BW (*p* = 0.001; *p* = 0.001), BMI (*p* = 0.013; *p* = 0.001), BFP (*p* = 0.001; *p* = 0.00), and WHR (*p* = 0.017; *p* = 0.00) in the AT+Vit D group compared to the AT and Vit D groups alone, respectively. Furthermore, the results showed a significant difference between AT and TS groups regarding BW (*p* = 0.001), BMI (*p* = 0.003), BFP (*p* = 0.018), and WHR (*p* = 0.024) (Table 3).

**Table 3:** Mean ± SD of anthropometric variables among the groups

Variables	AT + Vit D (*n* = 10)	AT (*n* = 10)	Vit D (*n* = 10)	C (*n* = 10)	*P*-Value
Age (years)	48.32 ± 2.23	47.13 ± 3.12	49.10 ± 1.2346	48.27 ± 2.17	0.540
Height (cm)	159.14 ± 2.13	161.17 ± 2.21	160.21 ± 2.28	162.12 ± 3.18	0.304
Duration of DM (year)	10.13 ± 3.23	9.10 ± 2.13	11.15 ± 1.89	10.25 ± 3.26	0.215
Body Weight (Kg)					
Before	75.11 ± 2.12	76.12 ± 3.14	74.18 ± 3.09	75.09 ± 2.17	
After	70.02 ± 1.97	73.14 ± 2.15	73.11 ± 2.19	76.35 ± 3.21	
P†	0.001*	0.021*	0.032*	0.028*	
Δ	−4.91 ± 0.15 ^**μ€β**^	−2.98 ± 0.99^**€β**^	−1.07 ± 0.9 ^**β**^	1.26 ± 1.04	0.001 ¥
BMI (kg/m^2^)					
Before	29.66 ± 1.25	29.30 ± 1.43	28.90 ± 1.23	28.57 ± 1.27	
After	27.65 ± 1.55	28.16 ± 1.29	28.48 ± 0.89	29.05 ± 1.12	
P†	0.011*	0.023*	0.046*	0.044*	
Δ	−2.01 ± 0.30 ^**μ€β**^	−1.14 ± 0.14 ^**€β**^	−0.42 ± 0.34 ^**β**^	0.48 ± 0.15	0.018 ¥
Body Fat Percent (%)					
Before	32.16 ± 1.56	31.11 ± 2.14	30.23 ± 1.56	29.76 ± 2.08	
After	28.11 ± 2.20	29.46 ± 1.69	29.55 ± 0.78	30.17 ± 1.78	
P†	0.001*	0.017*	0.035*	0.043*	
Δ	−4.05 ± 0.64 ^**μ€β**^	− 1.65 ± 0.45 ^**€β**^	− 0.68 ± 0.78 ^**β**^	0.41 ± 0.3	0.003 ¥
WHR					
Before	0.94 ± 0.01	0.93 ± 0.03	0.92 ± 0.04	0.92 ± 0.02	
After	0.88 ± 0.02	0.89 ± 0.02 ^**β**^	0.90 ± 0.02 ^**β**^	0.93 ± 0.03	
P†	0.012*	0.031*	0.001*	0.001*	
Δ	−0.06 ± 0.01 ^**μ€β**^	−0.04 ± 0.01 ^**€β**^	− 0.02 ± 0.02 ^**β**^	0.01 ± 0.01	0.001 ¥

*AT + Vit D* Aerobic training + Vitamin D supplement, *AT* Aerobic training group, *Vit D* Vitamin D supplement group, *C* the control group

Results from analysis of one-way analysis of variance (ANOVA), post-hoc Tukey’s test, and paired sample t-test

P†: indicating the results of paired sample t-test

*: Significant within-group differences between pre and post

¥: Significant within-group differences in Δ

β: Significant differences with C

μ: Significant differences with AT

€: Significant differences with Vit D

The results of between-group comparisons of the participants’ glycemic control, Vit D, biomarkers of inflammation, and oxidative stress are presented in Table 4. The t-test results showed significant differences in the mean of all the variables in the post-test compared to the pre-test. After 8 weeks, insulin, FBG, HOMA-IR, hs-CRP, MDA, glycated albumin, and 8-OHdG significantly decreased in all intervention groups, while these variables increased substantially in the C (Table 4). Serum 25-OH-Vit D, total nitrite, GSH, TAC, CAT, SOD, and GPX increased significantly after 8 weeks in all intervention groups except for C. After the intervention, AT+Vit D, AT, and Vit D all significantly lowered insulin, FBG, HOMA-IR, hs-CRP, MDA, glycated albumin, and urinary 8-OHdG. Serum 25-OH-Vit D, total nitrite, GSH, TAC, CAT, SOD, and GPX increased significantly compared to the C. Additionally, the AT+Vit D group had significantly lower insulin, FBG, HOMA-IR, hs-CRP, MDA, glycated albumin, and urinary 8-OHdG levels, and significantly higher serum 25-OH-Vit D, total nitrite, GSH, TAC, CAT, SOD, and GPX levels compared to the AT and Vit D groups. The results also showed a significant difference between the AT and Vit D groups in all the variables.

**Table 4:** Glycemic variables, vitamin D, inflammation, and oxidative stress biomarkers before and after the intervention in T2DM patients

Variables	AT + Vit D (*n* = 10)	AT (*n* = 10)	Vit D (*n* = 10)	C (*n* = 10)	*P*-Value
Insulin (μU/mL)					
Before	7.87 ± 0.29	7.59 ± 0.56	6.89 ± 0.23	6.73 ± 0.33	
After	5.54 ± 0.11	5.73 ± 0.18	6.20 ± 0.26	6.97 ± 0.23	
P†	0.011*	0.024*	0.031*	0.048*	
Δ	−2.33 ± 0.18 ^**μ€β**^	−1.86 ± 0.38 ^**€β**^	−0.69 ± 0.14 ^**β**^	0.24 ± 0.1	0.016 ¥
FBG (mg/dl)					
Before	169.76 ± 2.05	158.23 ± 2.41	150.18 ± 1.44	153.33 ± 2.54	
After	149.23 ± 1.67	146.14 ± 1.32	145.11 ± 2.23	157.39 ± 2.22	
P†	0.001*	0.001*	0.001*	0.003*	
Δ	−20.53 ± 0.38 ^**μ€β**^	− 12.09 ± 1.09 ^**€β**^	− 5.07 ± 0.79 ^**β**^	4.06 ± 0.32	0.001 ¥
HOMA-IR					
Before	3.30 ± 0.16	2.97 ± 0.26	2.55 ± 0.19	2.55 ± 0.24	
After	2.04 ± 0.23	2.07 ± 0.12	2.22 ± 0.31	2.71 ± 0.15	
P†	0.011*	0.019*	0.027*	0.047*	
Δ	−1.26 ± 0.07 ^**μ€β**^	−0.90 ± 0.14 ^**€β**^	−0.33 ± 0.12 ^**β**^	0.16 ± 0.09	0.026 ¥
serum 25-OH-Vit D (ng/mL)					
Before	22.22 ± 1.29	21.45 ± 2.57	23.17 ± 1.02	23.20 ± 3.41	
After	39.67 ± 0.84	28.26 ± 1.62	34.18 ± 1.24	22.70 ± 1.64	
P†	0.001*	0.002*	0.001*	0.111	
Δ	17.45 ± 0.45 ^**μ€β**^	6.81 ± 0.95 ^**€β**^	11.01 ± 0.22 ^**β**^	−0.50 ± 1.77	0.001 ^**¥**^
hs-CRP (mg/L)					
Before	2.91 ± 0.15	2.78 ± 0.11	2.85 ± 0.10	2.81 ± 0.16	
After	2.36 ± 0.17	2.43 ± 0.10	2.60 ± 0.12	2.96 ± 0.22	
P†	0.011*	0.027*	0.039*	0.045*	
Δ	−0.55 ± 0.02 ^**μ€β**^	−0.35 ± 0.01 ^**€β**^	− 0.25 ± 0.02 ^**β**^	0.15 ± 0.06	0.019 ¥
Total nitrite (μmol/L)					
Before	46.30 ± 2.09	48.08 ± 4.23	47.11 ± 2.21	47.50 ± 2.64	
After	62.60 ± 1.23	61.11 ± 2.03	54.16 ± 1.38	43.11 ± 1.25	
P†	0.001*	0.001*	0.001*	0.001*	
Δ	16.30 ± 0.86 ^**μ€β**^	13.03 ± 2.20 ^**€β**^	9.05 ± 0.83 ^**β**^	−4.39 ± 1.39	0.001 ¥
GSH (μmol/L)					
Before	472.21 ± 19.08	478.23 ± 20.25	489.13 ± 28.23	492.23 ± 21.08	
After	580.26 ± 10.36	540.17 ± 11.45	525.34 ± 18.21	478.90 ± 8.19	
P†	0.001*	0.001*	0.001*	0.015*	
Δ	108.05 ± 8.72 ^**μ€β**^	61.94 ± 8.80 ^**€β**^	36.21 ± 10.02 ^**β**^	−13.33 ± 12.89	0.001 ¥
MDA (μmol/L)					
Before	2.31 ± 0.03	2.15 ± 0.08	2.18 ± 0.08	2.11 ± 0.06	
After	1.89 ± 0.08	1.93 ± 0.05	2.04 ± 0.09	2.20 ± 0.03	
P†	0.002*	0.023*	0.032*	0.047*	
Δ	−0.42 ± 0.05 ^**μ€β**^	−0.22 ± 0.03 ^**€β**^	−0.14 ± 0.901^**β**^	0.09 ± 0.03	0.021 ¥
TAC (mmol/L)					
Before	910.80 ± 32.68	886.15 ± 19.23	890.33 ± 24.43	906.20 ± 27.13	
After	981.53 ± 21.46	936.36 ± 12.34	919.65 ± 14.06	887.12 ± 11.38	
P†	0.001*	0.001*	0.017*	0.036*	
Δ	70.73 ± 11.22 ^**μ€β**^	50.21 ± 6.89 ^**€β**^	29.32 ± 10.37 ^**β**^	−19.08 ± 15.75	0.001 ¥
Glycated albumin (%)					
Before	23.11 ± 1.08	22.10 ± 2.03	21.17 ± 2.13	22.43 ± 1.87	
After	18.12 ± 2.26	19.56 ± 1.34	20.08 ± 1.16	22.85 ± 0.74	
P†	0.014*	0.029*	0.037*	0.043*	
Δ	−4.99 ± 1.18 ^**μ€β**^	−2.54 ± 0.69 ^**€β**^	−1.09 ± 0.97 ^**β**^	0.42 ± 1.13	0.015 ¥
Urinary 8-OHdG (ng/mg creatinine)					
Before	13.22 ± 1.17	12.50 ± 0.58	12.33 ± 1.33	12.15 ± 0.36	
After	9.95 ± 0.67	11.14 ± 1.14	11.63 ± 1.09	12.45 ± 0.63	
P†	0.019*	0.021*	0.032*	0.046*	
Δ	−3.27 ± 0.50 ^**μ€β**^	−1.36 ± 0.56 ^**€β**^	−0.70 ± 0.24 ^**β**^	0.40 ± 0.27	0.018 ¥
CAT (U/mL)					
Before	82.13 ± 2.14	83.14 ± 3.17	84.23 ± 2.31	83.55 ± 1.30	
After	93.95 ± 3.67	89.16 ± 2.04	88.22 ± 1.36	81.64 ± 2.23	
P†	0.001*	0.001*	0.012*	0.038*	
Δ	11.82 ± 1.53 ^**μ€β**^	6.02 ± 1.13 ^**€β**^	3.99 ± 0.95 ^**β**^	−2.91 ± 0.93	0.001 ¥
SOD (U/mL)					
Before	11.23 ± 0.97	11.39 ± 0.87	11.55 ± 0.18	11.45 ± 0.54	
After	13.95 ± 1.07	13.14 ± 0.75	12.84 ± 0.32	10.40 ± 0.40	
P†	0.016*	0.023*	0.036*	0.041*	
Δ	2.72 ± 0.10 ^**μ€β**^	1.75 ± 0.12 ^**€β**^	1.29 ± 0.14 ^**β**^	−1.05 ± 0.15	0.021 ¥
GPX (mu/ml)					
Before	176.23 ± 6.23	181.11 ± 7.05	178.22 ± 4.08	180.19 ± 3.29	
After	194.14 ± 4.27	191.24 ± 2.34	186.12 ± 2.24	177.11 ± 2.24	
P†	0.001*	0.001*	0.001*	0.021*	
Δ	17.91 ± 1.96 ^**μ€β**^	10.13 ± 4.71 ^**€β**^	7.90 ± 1.84 ^**β**^	−3.08 ± 1.05	0.001 ¥

*AT + Vit D* Aerobic training + Vitamin D supplement, *AT* Aerobic training group, *Vit D* Vitamin D supplement group, *C* the control group

Results from analysis of one-way analysis of variance (ANOVA), post-hoc Tukey’s test, and paired sample t-test

P†: indicating the results of paired sample t-test

*: Significant within-group differences between pre and post

¥: Significant within-group differences in Δ

β: Significant differences with C

μ: Significant differences with AT

€: Significant differences with Vit D

**Fig. 2:**
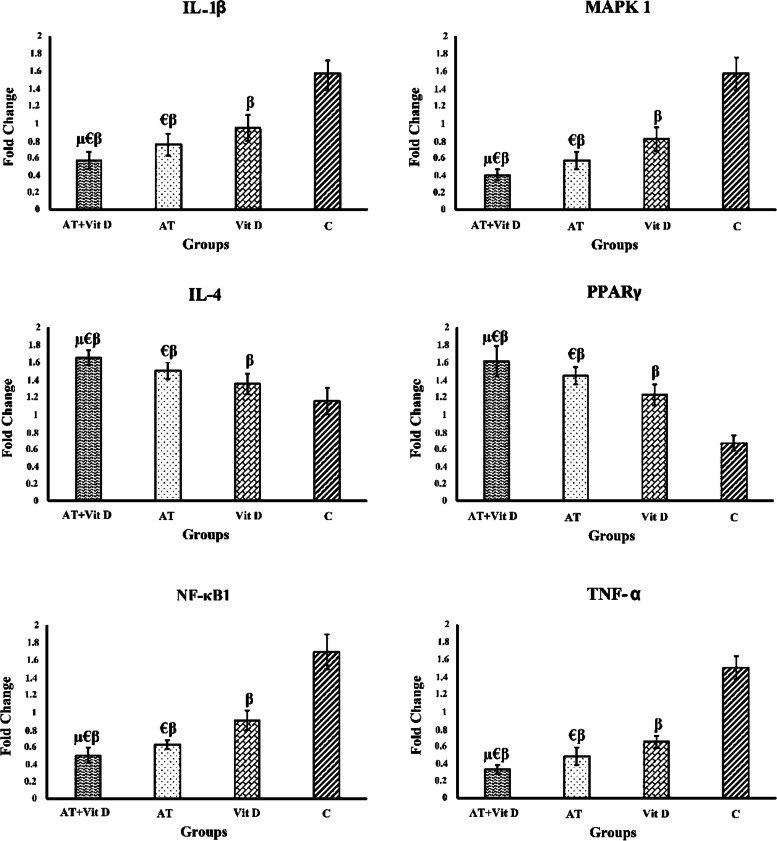
Effect of aerobic training and vitamin d supplementation on IL1-β, MAPK1, IL-4, PPAR-γ, NF-κB1, and TNF-α in patients with T2DM. IL-1β: Interleukin 1 Beta; MAPK1: Mitogen-Activated Protein Kinases; IL-4: Interleukin-4; PPAR-γ: Peroxisome Proliferator-Activated Receptor Gamma; NF-kβ: Nuclear Factor Kappa B; and TNF-α: Tumor Necrosis Factor Alpha. Results from analysis of one-way analysis of variance (ANOVA), post-hoc Tukey’s test. β: Significant differences with C. μ: Significant differences with AT. €: Significant differences with Vit D

Furthermore, AT+Vit D, AT, and Vit D downregulated IL1-β, MAPK1, NF-κB1 (p50), and TNF-α. They also upregulated IL-4, and PPAR-γ in T2DM patients compared to the C (Fig. 2). Based on the results, AT+Vit D significantly downregulated IL1-β (*p* = 0.009; *p* = 0.001), MAPK1 (*p* = 0.014; *p* = 0.001), NF-κB1 (p50) (*p* = 0.023; *p* = 0.001), and TNF-α (*p* = 0.024; *p* = 0.001) and significantly upregulated IL-4 (*p* = 0.032; *p* = 0.014), and PPAR-γ (*p* = 0.026; *p* = 0.001), compared to AT and Vit D, respectively. There were significant differences between AT and Vit D in the IL1-β (*p* = 0.033), MAPK1(*p* = 0.007), NF-κB1 (p50) (*p* = 0.001), and TNF-α (*p* = 0.029) and upregulated IL-4 (*p* = 0.027), and PPAR-γ (*p* = 0.018) (Fig. 2).

## Discussion

The present study results indicated reduced BW, BMI, BFP, and WHR in AT; however, this reduction was more significant when combining AT and Vit D supplementation. Regular AT improves body composition, insulin resistance, lipolytic enzyme expression, mitochondrial density, daily energy expenditure, and fat oxidation [34–38]. BW, BMI, and BFP were reduced in Vit D after 8 weeks based on the results. Hoseini et al. (2017) also documented reduced BW, visceral fat, and BMI in rats’ model of metabolic syndrome following high doses of Vit D [39]. These results could be due to the roles of Vit D, parathyroid hormone (PTH), and intercellular Ca++ in closing the GLUT4 channel and impairing the adipose tissue metabolic processes, glucose metabolism, and insulin function [38]. A further reduction in anthropometric indices following combined AT+Vit D, compared to AT or Vit D, might be related to the fact that Vit D and AT benefited the indices through different pathways or by affecting other downstream targets. In this study, 8 weeks of AT down-regulated gene expression of hs-CRP, IL-1β, TNF-α, IFN-γ, MAPK1, and NF-κB1 (p50), and upregulated IL-4 and PPAR-γ decreased oxidative damage (urinary 8-oHdG, glycated albumin, MDA, and hs-CRP). They increased antioxidant defense (GPx, SOD, CAT, TAC, GSH, and total nitrite) concomitantly, which is consistent with the recent results of Shephardet et al. (2000) [40], Gielenet et al., (2003) [41], Stewart et al., (2004) [42], Petersen et al., (2005) [20] Devrieet et al., (2008) [43], and de Oliveiret et al., (2012) [44]. Although the underlying mechanisms are unknown, the improvement of inflammatory and oxidative stress indicators generated by AT is well established, as shown in this study [40, 45, 46]. Based on our findings and those of other studies, underlining the importance of concurrent reductions in insulin resistance, BW, and, in particular, BFP in improving inflammatory and oxidative stress indicators could be a plausible mechanism [47, 48]. Moreover, it has been reported that AT mediates the myokines and adipokines secretion by targeting skeletal muscle and adipose tissue, which improves the inflammatory and oxidative stress biomarkers [20, 41]. As an endocrine tissue that expresses both NADPH oxidase and adipokines diminished BFP and obesity may simply give Redox homeostasis and a balanced inflammatory profile in T2DM patients, lowering the risk of chronic illnesses [43, 44, 49]. AT reduces inflammation by presenting multiplied physiological benefits such as reducing toll-like receptors and NF-κB1 (p50) expression. It also increases lipolysis, down-regulates leukocyte migration, and enhances angiogenesis [50, 51]. Based on the results, AT can suppress the mRNA expression of pro-inflammatory cytokines and enhance anti-inflammatory cytokines production. The present study results also showed a cross-talk between the enhancement of the oxidative defense system and the improvement of the inflammatory biomarkers following AT. However, some studies demonstrated no change in the mRNA expression of some inflammatory biomarkers following long-term AT [52, 53]. Thus, it could be hypothesized that combining AT and Vit D might be a powerful strategy for T2DM patients to improve inflammatory and oxidative stress biomarkers.

**Fig. 3:**
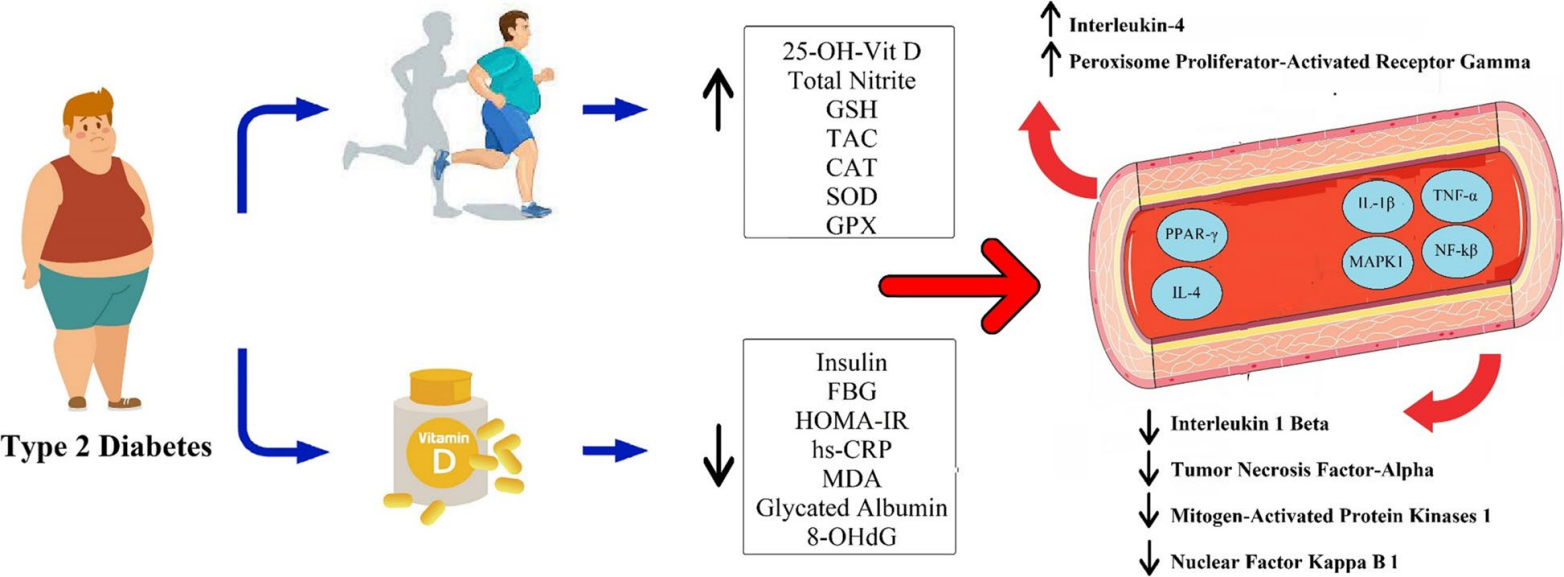
Role of aerobic training and vitamin D on inflammatory biomarker gene expression and oxidative stress in type 2 diabetes mellitus. GSH: Total Glutathione; TAC: Total Antioxidant Capacity; CAT: Catalase; SOD: Superoxide Dismutase; GPX: Glutathione Peroxidase; FBG: Fasting Blood Glucose; hs-CRP: High Sensitivity C-Reactive Protein; MDA: Malondialdehyde; 8-OHdG: Urinary 8-hydroxydeoxyguanine; IL-1β: Interleukin 1 Beta; TNF-α: Tumor Necrosis Factor-Alpha; MAPK1: Mitogen-Activated Protein Kinases 1; NF-κB1: Nuclear Factor Kappa B 1; IL-4: Interleukin-4; PPAR-γ: Peroxisome Proliferator-Activated Receptor Gamma

To the best of our knowledge, data on AT and Vit D supplementation on inflammation-related gene expression and oxidative stress biomarkers among T2DM patients are limited. The results revealed 8 weeks of combined Vit D and AT downregulated hs-CRP, IL-1β, TNF-α, MAPK1, and NF-κB1 (p50) gene expressions. Also, upregulated IL-4 and PPAR-γ, decreased oxidative damage (urinary 8-oHdG, glycated albumin, MDA, and hs-CRP), and increased antioxidant defense (GPx, SOD, CAT, TAC, GSH, and total nitrate) were observed. Based on current evidence, Vit D supplementation reduced hs-CRP [54–56]. In line with our findings, Willis et al. (2012) documented an inverse relationship between TNF-α and Vit D levels [57], Schleithoff et al. (2006) reported the suppression of TNF-α production following 9 months of Vit D supplementation [58], Müller et al., (1992) showed suppressed IL-1β and TNF-α [59], and Irani et al., (2015) showed decreased TGF-b1 after 8 weeks of Vit D administration (50,000 IU/week) [60]. Furthermore, some cross-sectional studies demonstrated decreased MAPK1 gene expression [61, 62], suppressed NF-κB1 (p50) [63], and increased PPAR-γ expression [39] induced by 1,25-(OH)2D3. However, contradictory findings show no significant changes in various inflammation and oxidative stress indicator after Vit D supplementation in healthy overweight and obese people, which could be related to the wide range of supplementation dosages, periods, and subjects’ conditions [28, 64, 65]. Chronic inflammation in T2DM is associated with progressing cardiovascular risk factors and is also one of the leading players in insulin function, resulting in hyperinsulinemia [66, 67]. Hyperinsulinemia resulting from chronic inflammation is related to consequent hyperandrogenism, insulin resistance, oxidative stress, and cardiovascular events in the T2DM population [67]. Vit D might decrease clinical and metabolic symptoms in T2DM by affecting oxidative stress and inflammatory cytokines, given Vit D’s antagonism to calcium which plays a crucial role in inflammatory responses [68]. This study also showed that Vit D supplementation downregulated the MAPK1 gene expression. Moreover, the MAPK1 signaling pathway has known to be crucial in the transcription and translation of biomarkers of inflammation and oxidative stress [69, 70]. Jafari et al. (2021) reported that Vit D supplementation along with exercise modulates antioxidant defense and the expression of antioxidant genes [71]. Vit D is also considered an antioxidant that might cause the expression of several genes involved in the antioxidant defense system, including GPX, GSH, CAT, SOD, and the suppression of NADPH oxidase [72, 73]. Besides, increasing Vit D levels in individuals with insufficient Vit D levels (trained and untrained) has improved antioxidant capacity [74]. Overall, Vit D deficiency has adverse effects on antioxidant capacity, and Vit D supplementation upregulates the activity of SOD and CAT [75] (Fig. 3).

### Strengths and limitations

This study’s strength was using a randomized, placebo-controlled, single-blind trial with a low dropout rate and evaluating the gene expression alterations in human subjects. Also, this study was conducted in the winter; thus, the seasonal changes played a minor role. However, considering the small sample size, it is premature to conclude a definitive answer. Also, using a self-reported questionnaire to measure and control daily diet and physical activity and not evaluating the alterations of transcriptional factors such as AP-1 and Nrf2/HO-1 signaling were among the limitations of the present study that is suggested to be considered in future studies.

## Conclusions

This study showed the potential molecular benefits of combined AT+Vit D on improving anthropometric indices, inflammation, and oxidative stress biomarkers in T2DM patients. Further studies are needed to find the best dose and training approach.

## Data Availability

The datasets used and/or analyzed during the current study are available from the corresponding author on reasonable request.

## Abbreviations

AT: Aerobic Training
Vit D: Vitamin D
T2DM: Type 2 Diabetes Mellitus
GSH: Total Glutathione
TAC: Total Antioxidant Capacity
CAT: Catalase
SOD: Superoxide Dismutase
GPX: Glutathione Peroxidase
FBG: Fasting Blood Glucose
hs-CRP: High Sensitivity C-Reactive Protein
MDA: Malondialdehyde
8-OHdG: Urinary 8-hydroxydeoxyguanine
IL-1β: Interleukin 1 Beta
TNF-α: Tumor Necrosis Factor-Alpha
MAP K1: Mitogen-Activated Protein Kinases 1
NF-κB 1: Nuclear Factor Kappa B 1
IL-4: Interleukin-4
PPAR-γ: Peroxisome Proliferator-Activated Receptor Gamma.
